# On Using Collocation in Three Dimensions and Solving a Model Semiconductor Problem

**DOI:** 10.6028/jres.100.049

**Published:** 1995

**Authors:** J. F. Marchiando

**Affiliations:** National Institute of Standards and Technology, Gaithersburg, MD 20899-0001

**Keywords:** boundary value problems, collocation, Poisson’s equation, semiconductor, three-dimensional

## Abstract

A research code has been written to solve an elliptic system of coupled nonlinear partial differential equations of conservation form on a rectangularly shaped three-dimensional domain. The code uses the method of collocation of Gauss points with tricubic Hermite piecewise continuous polynomial basis functions. The system of equations is solved by iteration. The system of nonlinear equations is linearized, and the system of linear equations is solved by iterative methods. When the matrix of the collocation equations is duly modified by using a scaled block-limited partial pivoting procedure of Gauss elimination, it is found that the rate of convergence of the iterative method is significantly improved and that a solution becomes possible. The code is used to solve Poisson’s equation for a model semiconductor problem. The electric potential distribution is calculated in a metal-oxide-semiconductor structure that is important to the fabrication of electron devices.

## 1. Introduction

As the features of electron devices are made smaller and less isolated, it becomes increasingly important to use three-dimensional models to characterize and understand the behavior of the semiconductor devices and to monitor and control the material processes that affect fabrication.

Accordingly, the numerical solution of three-dimensional models becomes increasingly important as well [1–6]. To assist in these aims, a research code has been written to solve a class of simple boundary value problems that involve an elliptic system of coupled nonlinear partial differential equations (PDEs) of conservation form on a rectangularly shaped three-dimensional domain.

The code uses the method of collocation of Gauss points with a set of tricubic Hermite piecewise continuous polynomials as basis functions. The elliptic system of *N* PDEs is of the form:

$$\nabla \cdot (a_{i}\nabla u_{i})=f_{i},\;i=1,2\ldots ,N,$$ (1)

where

$$\begin{array}{l} u_{i}=u_{i}\;(x),\\ x=\left(x_{1},\;x_{2},\;x_{3}\right)=(x,\;y,\;z)\;\in \;D,\\ u=\left(u_{1},\;u_{2},\ldots ,u_{N}\right),\\ a_{i}=a_{i}(x,\;u),\\ f_{i}=f_{i}(x,\;u,\;\partial u/\partial x,\;\partial u/\partial y,\;\partial u/\partial z), \end{array}$$

are defined on a single rectangularly shaped three-dimensional domain *D*. Each solution *u_i_* is required to satisfy a generally nonlinear but linearizable boundary condition of the form:

$$g_{i}(x_{k},\;u,\;\partial u/\partial x,\;\partial u/\partial y,\;\partial u/\partial z)=0,\;x_{k}\in \;\partial D,$$ (2)

where *k* indexes collocation and mesh points on the domain boundary ∂*D*. While these forms are quite general, it is well-known that a number of conditions on the functions *a*, *f*, and *g* must be satisfied for a solution ***u*** to exist. While these conditions are of great importance, they are not discussed here, but are discussed elsewhere [7–9], and it is assumed that they are satisfied here. Further discussion regarding existence, uniqueness, stability, well-posedness, definiteness, the collocation method, and methods of solution may be found in the literature [7–17].

Few system solvers seem to be generally available today because of the inherent difficulty in solving nonlinear problems, the large resource requirements for three dimensions, and the need to monitor and control the local refinement of the discretization mesh that is needed to maintain numerical stability and provide favorable rates of convergence. These solvers tend to use the linear finite element method (FEM) because of the simplicity of the basis functions and the ease in which the elements can be made to cover domains of complicated shape. And, while the more sophisticated system solvers may make available a number of powerful features to the user, e.g., a geometric modeller to help form and discretize the domain, the packaged solvers may also restrict the form of the PDEs that may be solved.

This restriction can become a problem when the allowed form is not sufficiently general to handle the kind of nonlinearies that the user wants to study, as was found to be the case with one commercially available FEM system solver [18] and the semiconductor device system of equations. One may hope that later versions of these packages would make it easier for their PDEs to be specified via some user-supplied subroutines like that found in B2DE [10] or PLTMG [19] but in three dimensions, and that they would incorporate some form of the multigrid algorithm as well.

Another reason that the linear finite element method is likely to be used in a solver is that the collocations equations are known to be difficult to solve [12,14–17]. Finding a solution to the collocation equations usually requires a direct solution by Gauss elimination, but fill-in, which degrades sparsity, becomes a problem for large systems of equations. Iterative methods have been applied [9,15,16], but this has usually involved scalar problems, not systems. Furthermore, collocation software usually implement tensor product meshes to partition the domain. While this expedites code development, it limits the shape of the domains and becomes less efficient for domains of higher dimensionality even with an adaptive mesh refinement capability or strategy.

Fortunately, much progress has been made in solving large sparse linear systems of equations by using iterative methods [20–29]. To help solve the large linear system of collocation equations, it has been convenient to use software packages like QMRPACK [20–22] and LSQR [23, 24]. When such solvers are applied directly to the collocation equations, it has been found that the convergence may be slow or nonexistent, even with preconditioners like the dual Threshold Incomplete LU factorization (ILUT) and the Symmetric Successive Overrelaxation (SSOR) methods [22, 26]. But, when the matrix of collocation equations is duly modified by using a scaled block-limited partial pivoting procedure of Gauss elimination [27], it is found that the rate of convergence of the iterative method is significantly improved and that a solution becomes possible.

This paper presents those considerations that have been found to be important when developing software that uses the collocation method to solve a class of simple boundary value problems in three dimensions. Section 2 presents an overview of these considerations; Sec. 3 presents the implementations and software requirements; and Sec. 4 presents an example of this implementation by formulating Poisson’s equation for a model semiconductor problem.

## 2. Overview of Considerations

Approximating a solution ***u*** of Eqs. (1) and (2) involves, in part, the following considerations [12]: (i) partitioning the domain *D* with a finite-element mesh *Ω*, (ii) determining a piecewise continuous polynomial approximation *U* defined over the partition *Ω*, (iii) linearizing the system of PDEs and boundary conditions to establish the collocation equations, (iv) solving the linear system of collocation equations, (v) updating the solution and monitoring the iterative procedure to convergence, and (vi) monitoring local and global error estimates of the solution *U* on *Ω* to provide a feedback mechanism to adaptively refine the partition on *D* to reduce the local and global error of the solution to within some predetermined or threshold value. While these considerations are quite general in nature, this paper discusses only those features that have been implemented into the current version of the research code. They include items (i–v), but not (vi), with particular attention given to item (iv). While the implementation of item (vi) is very important and is a topic that has received and continues to receive much attention in the literature [7], no attempt is made to consider it here, but is left to future work.

Because Eqs. (1) and (2) may be nonlinear, a conventional method of solution is used. The equations are solved iteratively with Newton’s method. The system of nonlinear equations are linearized in the usual manner; see Appendix A. The system of linear equations are solved by an iterative procedure that uses QMRPACK [22], a software package that implements both the ILUT and SSOR preconditioners, the look-ahead Lanczos algorithm, and the quasi-minimal residual method. While it is known [12,14–17] that the collocation equations are difficult to solve, i.e., usually requiring a direct solution by Gauss elimination, it is found that when the matrix of the collocation equations is duly modified by using a scaled block-limited partial pivoting procedure of Gauss elimination, the rate of convergence of the iterative procedures is significantly improved, and a solution becomes possible.

The idea behind the scaled block-limited partial pivoting procedure is to impart additional orthogonalization into the initial or usual set of collocation equations, and this, of course, reduces the amount of work that is needed to find a solution by the preconditioner and the Krylov method [26] that is used in iterative solvers like QMRPACK and LSQR. The term *block-limited* refers to limiting the pivoting procedure to the set of collocation equations that are formed by the collocations points that are nearest to and positioned about or on a given mesh point. There are eight such collocation points for each mesh point of the three-dimensional domain, and thus there are eight collocation equations per mesh point per solution component or PDE that is being considered. Here, the interest is in determining the independent variables of the local collocation vector of the Newton step; see Appendix B.

Full pivoting of Gauss elimination is applied to the set of eight equations so that the matrix of coefficients of the shuffled variables of the local collocation vector becomes upper triangluar. The pivot elements are determined in the usual manner, but where the rows under consideration are kept scaled to unit *L*_2_ seminorm. Rescaling equalizes the rows during pivot selection, and this stabilizes the numerics, as is well-known. This procedure is convenient, because it automatically orders the equations and the variables without special consideration being given to the boundary conditions. This is important when one later uses an iterative solver that does no partial pivoting. After the entire matrix of collocation equations is duly modified, the variables may be rescaled to improve the behavior of iteration. Currently, the variables are scaled such that the matrix product of the matrix and its transpose have unit diagonal elements, i.e., (*J^T^J*)*_ii_* = 1, where *J* refers to the collocation matrix, and the presence of two like indices does not imply summation.

The matrix of collocation equations is then submitted to the linear equation solver QMRPACK with ILUT preconditioning. While the ILUT of QMRPACK allows the user to specify left, right, or left and right preconditioning, and the number and tolerance of fill-in per row, these are currently set as left and right, 0, and 1×10^–6^, respectively. These values are not necessarily optimal.

Following the iterative solution of the system of linear equations, i.e., the Newton step solution ***v***, the current solution ***u*** of the system of PDEs is then updated by the Newton step, i.e., ***u***^(k+1)^ = ***u***^(k)^ + ***v***^(k)^, where *k* indexes the outer loop of iterations.

At the end of each outer loop of iterations, a decision is made regarding the convergence properties of the last iteration. If the *L*_2_ seminorm of the residual falls below some predetermined value or if the *L*_2_ seminorm of the residual fails to be reduced in five iterations, then the outer loop of iterations is interrupted, the solution is breakpointed, and control is returned to the calling program. Otherwise, the outer loop of iterations is continued.

## 3. Implementation

As mentioned in Ref. [12], the implementation of the collocation method to solve a system of PDEs involves a number of considerations. They include defining: the array allocations to satisfy the workspace requirements; the problem by specifying the domain, the PDEs, and the boundary conditions; the discretization of the domain; and the initial value solution. These are input to the collocation solver.

The collocation solver is then used to: form the collocation vector (see Appendix B), form the collocation equations, and solve the collocation equations by iteration. The solution procedure involves a nest of iterations, an inner loop for the linear problem and an outer loop for the nonlinear problem.

Regarding software development, most of the code was written in standard Fortran-77, except where a few variable and subroutine names were allowed to exceed the six character length limit. The maximum length of a subroutine name was 10 characters, e.g., prefixing qmr_ to the names of two subroutines that were taken and modified from QMRPACK. Lengthy names were chosen for sake of clarity of purpose or origin. While most variable/subroutine names are of six characters or less, the few lengthy names ought to pose no problem with the current generation of compilers.

### 3.1 Specifying the Array Allocations

The array allocations used in the program are of two kinds, those that do and those that do not depend on the four parameters that determine the maximim allowed size of the collocation vector. The collocation vector is discussed further in Appendix A. The four parameters are: mpde, mxgrid, mygrid, mzgrid; and they refer to the number of solution components or PDEs to be solved, the number of grid points associated with the *x*-axis, the number of grid points associated with the *y*-axis, and the number of grid points associated with the *z*-axis, respectively. These must be set before the program is compiled and linked. The four parameters are set in a file named defnit.

A few other definition files use these parameters to define other parameters and to allocate arrays and named common blocks. The files have suggestive names like: defnit.2, stackg., stackj., stack5., stack6., stackp. [1–3], stackw.2, where stackg contains arrays for the grid discretization, stackj contains arrays for the Jacobian, stackp contains arrays for the preconditioners and QMRPACK, stackw contains arrays for workspace for LSQR, etc., and where the brackets refer to the usual Unix convention restricting wild-card searches over a range of characters. These files are intended to remain unmodified by the user. Most of the arrays are assigned to a named common block. These arrays are made available to the subroutines via the Fortran INCLUDE statement. EQUIVALENCE statements occur in files stackp.1 and stackw.2. Because the common blocks and EQUIVALENCE statements are in definition files, it is a relatively simple task to change the names of named common blocks, if necessary.

While passing arguments via common blocks instead of subroutine argument lists restricts the generality of the subroutines, there is merit in simplifying the argument lists especially during the initial phases of code development. Further refinements are possible, but these are left to the discretion of the user.

### 3.2 Specifying the Domain and the PDEs

The domain is a rectangularly shaped three-dimensional block volume region where the coordinate system is Cartesian and each of the six boundaries has a surface normal vector that is parallel with a coordinate axis. The domain is discretized by the discretization of the axes. The finite-element mesh of an axis is specified by an array of values that are ordered as monotonically increasing. Hence, the domain is specified by using three arrays, one for each axis.

The boundary value problem defined by Eqs. (1) and (2) is specified via the function terms *a, f*, and *g*. These terms are made available to the collocation solver by using three user-supplied external subroutines, one for each term *a*, *f*, and *g* (see Appendix A). This was inspired by the design of B2DE [10]. Because one subroutine is used to make all of the calls to the subroutine of the function term *a*, it is relatively straightforward to modify the program to form the collocation matrix of a more general class of second-order differential operator, e.g.,

$$\sum_{\alpha =1}^{3}\sum_{\beta =1}^{\alpha }a_{i\alpha \beta }(x,u,\partial u/\partial x_{1},\;\partial u/\partial x_{2},\;\partial u/\partial x_{3})\frac{\partial^{2}u_{i}}{\partial x_{\alpha }x_{\beta }},$$ (3)

which would be needed for non-Cartesian coordinate systems. (Of course, this could affect the convergence properties of the iterative methods that are used to solve the linear algebra problem of the resulting set of collocation equations, perhaps by requiring more robust preconditioning.) Regarding the subroutine of the function term *g*, an integer variable *isw* is used in the argument list to specify the direction of the surface normal vector. The magnitude of *isw* determines the coordinate axis, where values {1,2,3} refer to {*x*_1_,*x*_2_,*x*_3_} axes, respectively. The sign of *isw* determines the direction of the surface normal. No provision is made for periodic boundary conditions.

### 3.3 The Initial Value Solution

The initial value solution is passed to the collocation solver as an array of point function values on the finite-element mesh. While the array is of dimension one, the elements are organized according to the Fortran convention dimension statement with the argument *uu*(*nx*,*ny*,*nz*,*nu*), where

*uu*: refers to the name of the array,
*nx*: refers to the number of *x*-axis grid points,
*ny*: refers to the number of *y*-axis grid points,
*nz*: refers to the number of *z*-axis grid points, and
*nu*: refers to the number of solution components or PDEs to be solved.

When the array is passed to the collocation solver, the collocation solver uses subroutines DB3INK to fit the point function with a tensor product of one-dimensional B-splines, and DB3VAL to evaluate the partial derivatives that are needed to form the initial collocation vector. B3INK/DB3INK and B3VAL/DB3VAL are based on the methods of de Boor [30] and are distributed by GAMS [31].

### 3.4 The Argument List

The subroutine name of the collocation solver is ESPDESC, an acronym for Elliptic System of PDEs Solved by Collocation. The argument list of the subroutine is (*nx, ny, nz, nu, xx, yy, zz, uu, aa, ff, gg, lu, iprint, init, iout, nwrk, wwrk*),where

*nx*: refers to the number of values in array *xx*,
*ny*: refers to the number of values in array *yy*,
*nz*: refers to the number of values in array *zz*,
*xx*: refers to the array of *x*-axis grid point values,
*yy*: refers to the array of *y*-axis grid point values,
*zz*: refers to the array of *z*-axis grid point values,
*uu*: refers to the array of the initial value solution,
*aa*: refers to the user-supplied external subroutine of *a*,
*ff*: refers to the user-supplied external subroutine of *f*,
*gg*: refers to the user-supplied external subroutine of *g*,
*lu*: refers to the array that determines which PDEs are active to identify which solution components undergo variation during iteration,
*iprint*: refers to the level of printing diagnostic messages that ranges from 1 for minimal to 6 for maximal output,
*init*: refers to the initialization switch that signals the start or continuance of a calculation,
*iout*: refers to the unit variable for diagnostic output,
*nwrk*: refers to the size of array *wwrk*, and
*wwrk*: refers to the workspace array used by DB3INK to spline fit the point function of the initial value solution.

Further discussion of the arguments may be found in the prologue of the subroutine. Following a normal return from the subroutine, the calculated solution may be found in three places: the collocation vector that is listed in the file stackg., the breakpoint solution that is saved in file x.bkpt, and the point function values that are returned in array *uu*.

## 4. The Model Semiconductor Problem

The three-dimensional collocation solver was developed to model the measurements by a scanning capacitance microscope of a semiconductor wafer that contains an ion-implanted impurity region as is used in the fabrication of electron devices. The measurement process involves placing a small metal probe-tip near the surface of a uniformly thin insulator layer that blankets the surface of the doped semiconductor substrate. A small bias voltage with an even smaller alternating current component voltage is then imposed on the probe-tip [32]. The component voltage displaces the electron and hole distributions in the semiconductor slightly away from the biased steady-state values. The differential capacitance is (Δ*Q*/Δ*V*), and a measurement of the capacitance (impedance) in the circuit of the probe and the sample is used to infer the doping concentration in the semiconductor region that is near the probe-tip. Such information can be useful in monitoring a fabrication process. To demonstrate the utility of the three-dimensional collocation solver, the presentation here is limited to that of estimating the size of the region in the semiconductor that is perturbed by the probe-tip bias.

The electron and hole distribution in the semiconductor region is determined by solving Poisson’s equation for the distribution of the electric potential function,

$$\nabla \cdot \left(\varepsilon_{r}\nabla \psi \right)=-\left(q/\varepsilon_{0}\right)\left(N_{d}-N_{a}+p-n\right),$$ (4)

where *q* refers to the magnitude of the elementary charge on the electron (1.602×10^−19^ C), *ε*_0_ refers to the relative permittivity of free space (8.854×10^−18^ F/μm), *ε*_r_ refers to the relative dielectric constant of the material (11.9 for Si, 3.9 for SiO_2_, and 1.0 for air), *N*_d_(***x***) refers to the number density of the ionized donor impurity distribution (μm)^−3^, *N*_a_(***x***) refers to the number density of the ionized acceptor impurity distribution (μm)^−3^, *n*(***x***) refers to the number density of the mobile electron distribution (μm)^−3^, and *p*(***x***) refers to the number density of the mobile hole distribution (μm)^−3^, and *ψ*(***x***) refers to the electric potential distribution (*V*). For nondegenerate semiconductors with a parabolic band structure at thermal equilibrium, the number densities *n* and *p* are related to the potential *ψ* via Boltzmann statistics,

$$n=n_{i}\exp(+(\psi +\psi_{0})/\phi ),$$ (5)

$$p=n_{i}\exp(-(\psi +\psi_{0})/\phi ),$$ (6)

where

ϕ=kT/q (7)

refers to the thermal voltage, *T* refers to the temperature (300 K), *k* refers to the Boltzmann constant (8.617×10^−5^ eV/K), *ψ*_0_ refers to a constant that establishes the zero of the potential (Fermi energy), and *n*_i_ refers to the intrinsic carrier concentration (1.379×10^−2^ (μm)^−3^ [33].

The electric potential in the insulator region is governed by Laplace’s equation, that is of the same form as Eq. (4), except that the source terms on the right-hand side of the equation are zero. At the insulator-semiconductor boundary, the potential is continuous, and the discontinuity of the normal component of the electric displacment vector depends on the trapped interfacal charge. Letting the interfacial charge density be zero, and letting the semiconductor region and the insulator region be labeled by 1 and 2, respectively, the boundary conditions at the interface are

$$\psi_{1}=\psi_{2},$$ (8)

and

$$\varepsilon_{1}\frac{\partial \psi_{1}}{\partial v}=\varepsilon_{2}\frac{\partial \psi_{2}}{\partial v},$$ (9)

where *v* refers to an outward normal.

The configuration of the sample is that of a two-layered structure. The thick layer is the semiconducting region and is made of crystalline silicon (Si) with a low concentration of ionized dopant impurities. The thin layer is the insulating region and is made of amorphous silicon dioxide (SiO_2_). While this is a model structure, it ought to be realized that an actual fabrication process may involve oxidizing the semiconductor surface either before or after placing the photolithographic line mask on the wafer, ion-implanting the wafer, and then removing the line mask. Furthermore, an annealing process usually follows the implantation process to remove the displacement damage of implantation and to activate or ionize the implanted dopant impurities. These processes can displace the initial implanted distribution relative to the surface, and this is important in real applications. However, for the calculations presented here, it is assumed that the processing is such that these effects are small and can be ignored. The ions are implanted into bare silicon, and an oxide layer is placed on the surface of the implanted silicon.

**Fig. 1:**
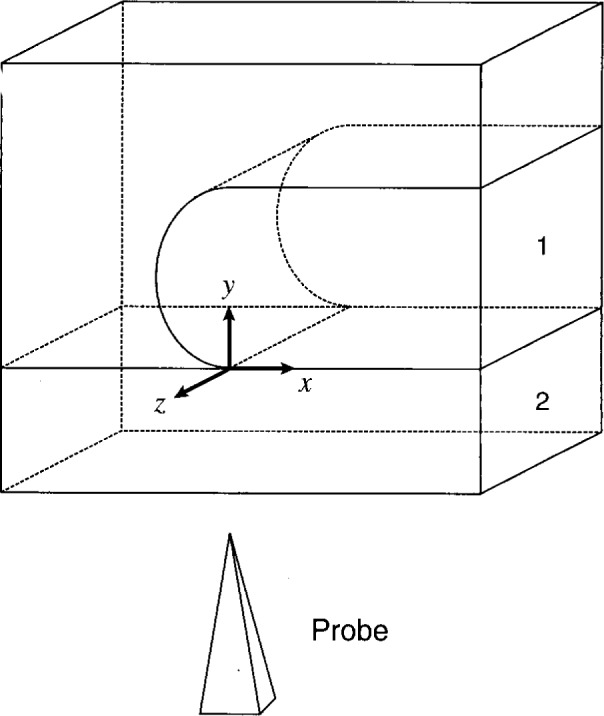
The geometry of the model sample structure. *D*_1_ refers to the semiconductor region (0 ≤ *y* ≤ 1), and *D*_2_ refers to the insulator region (−0.02 ≤ *y* ≤ 0).

The geometry of the sample structure is presented in Fig. 1. The unit of length is expressed in μm. The coordinate system is chosen such that the domain *D* containing both the insulator and the semiconductor regions be given by (−1 ≤ *x* ≤ 1), (−0.02 ≤ *y* ≤ 1), (−0.2 ≤ *z* ≤ 0). The semiconducting region *D*_1_ is that where (*y* ≥ 0), and the insulating region *D*_2_ is that where (*y* ≤ 0). The (*y* = 0) plane forms the SiO_2_/Si interface boundary and is the plane through which ion-implanted dopants were implanted. The *y*-axis is directed into the substrate region. The *z*-axis is aligned parallel with the earlier line mask edge. The *z*-axis is a direction of translational invariance, i.e., before the introduction of the probe-tip as specified by the boundary conditions. The *x*-axis is directed from the unimplanted masked region to the implanted unmasked region.

**Fig. 2:**
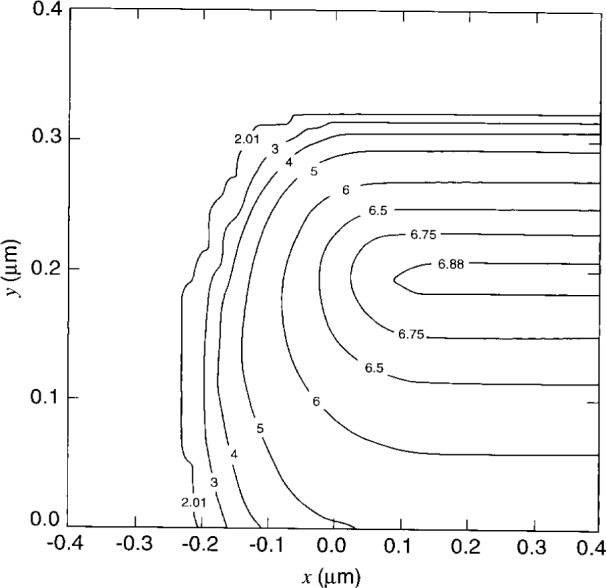
A contour plot of the logarithm of the concentration of the ion-planted boron dopant distribution near a line mask edge, as determined by TRIM Monte Carlo calculation [34]. The direction normal to the plane of the figure is parallel with the line mask edge and is a direction of translational invariance. The implanted surface is the (*y*= 0) plane, where the masked region is (*x* ≤ 0).

**Fig. 3:**
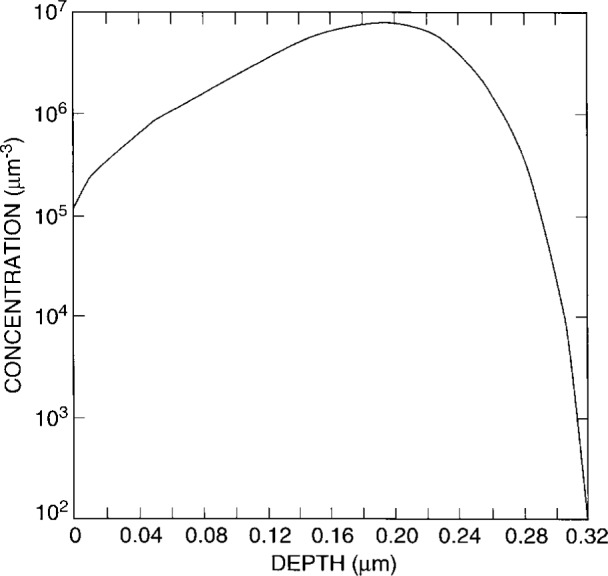
A profile of the boron concentration in silicon as a function of depth normal to the surface far from a mask edge, (*x* > 0.69 μm), as determined by a TRIM Monte Carlo Calculation [34]. The implanted surface is the (*y* = 0) plane.

The semiconductor region is uniformly doped with arsenic; the donor concentration is 5×10^3^ (μm)^−3^. The ion-implanted impurity doping distribution of boron acceptors into silicon near a mask edge is determined by a Monte Carlo calculation [34]. The implant voltage is 50 keV, and the number of sampling events or histories is 1×10^5^. The dose is then rescaled to 1×10^6^ (μm)^−2^; the peak acceptor concentration is found to be 8×10^6^ (μm)^−3^. And finally, the dopant impurities are fully ionized. The ion-implanted dopant distribution is presented in Figs. 2 and 3. Figure 2 presents a contour plot of the logarithm of the concentration of the ion-implanted boron dopant distribution near the line mask edge, where the direction normal to the plane of the figure is parallel with the earlier line mask edge and is a direction of translational invariance. Figure 3 presents a profile of the concentration of the ion-implanted boron dopant distribution as a function of depth into the substrate in a direction normal to the surface and in a place far from the line mask edge that is exposed to the ion-implantation beam.

The characteristic size of the probe-tip is 0.06 μm. The probe-tip is modeled as a square patch that is placed on the outer surface of the oxide, the (*y* = −0.02) plane. The patch is centered generally about a point with some variable value *x*_p_ but with fixed value (*z* = 0). The patch is oriented such that the (*z* = 0) plane is a symmetry reflection plane. For the calculations presented here, (*x*_p_ = 0), thus, the patch occupies the region where (−0.03 ≤ *x* ≤ 0.03) and (−0.03 ≤ *z* ≤ 0). The probe-tip bias voltage is specified with the Dirichlet boundary condition, (*ψ* = 3).

The back plane (*y* = 1) of the semiconductor is grounded with the Dirichlet boundary condition, (*ψ* = 0). Requiring charge neutrality at the grounded plane sets *ψ*_0_. The remaining outer surfaces have Neumann boundary conditions, where the normal derivative of *ψ* is set to zero.

This problem involves two subdomain regions, the semiconductor region *D*_1_ and the insulator region *D*_2_. The method of solution involves cycling through the subdomain regions, where the subdomains are treated individually and in ordered succession, and where the PDEs of each subdomain are solved. The boundary conditions are specified in a manner that lead to their becoming satisfied along the interface as the iterations proceed unto self-consistency, i.e., convergence.

**Fig. 4:**
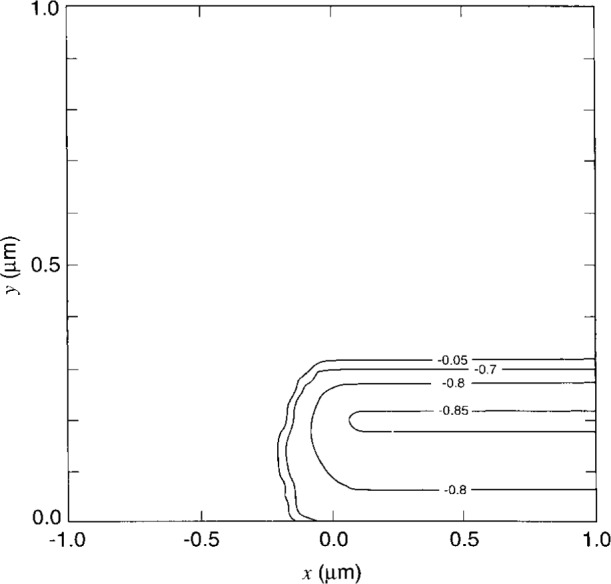
A contour plot of the electric potential distribution of the initial value or charge neutral solution in the semiconductor region near the mask edge in the (*z* = 0) plane.

**Fig. 5:**
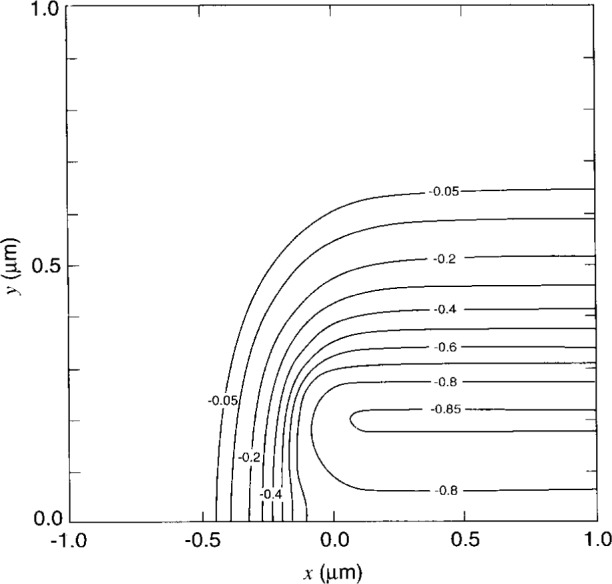
A contour plot of the electric potential distribution of the calculated unbiased steady-state or equilibrium solution in the semiconductor region near the mask edge in the (*z* = 0) plane.

**Fig. 6:**
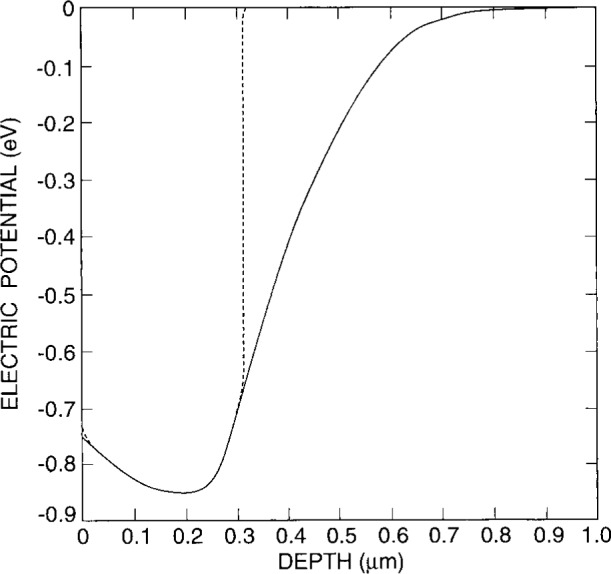
A profile of the electric potential of the initial value or charge neutral solution (dashed line) and the unbiased steady-state or equilibrium solution (solid line) in the semiconductor region, as a function of depth normal to the surface far from a mask edge (*x* > 0.69 μm).

One procedure that is found to converge is to start with the semiconductor region. The initial value solution is set to the charge neutral solution, and the interface boundary condition is specified as Neumann where the normal derivative of the potential is set to zero, i.e., charge is not allowed to escape through the interface boundary. Poisson’s equation is then solved in the semiconducting region. Figure 4 presents the two-dimensional distribution (*z* = 0 plane) of the electric potential of the initial value or charge neutral solution. Figure 5 presents the two-dimensional distribution (*z* = 0 plane) of the electric potential of the calculated unbiased steady-state or equilibrium solution. Figure 6 presents the profiles of the electric potential of both the initial value or charge neutral solution and the unbiased steady-state or equilibrium solution as a function of depth (*y*) from the interface and in an implanted region far from the mask edge, (*x* = 1). The negative values of the distribution are in accord with Eq. (5), where the material is *p*-type due to the implanted boron, an acceptor.

The initial value solution of the insulating region is then found by simply translating along *y* the solution at the interface boundary. Letting the interface boundary condition be Dirichlet by Eq. (8), Laplace’s equation is then solved in the insulating region. This provides a newly determined value of the upper/lower bound of the normal derivative of the solution in the semiconductor at the interface, as found by using Eq. (9). The interface boundary condition for the semiconducting region is then updated by averaging this newly determined value and the previous value of the normal derivative of the solution in the semiconducting region, so that one may again solve Poisson’s equation in the semiconductor region. This completes one cycle of the iterative procedure [29].

The convergence of this procedure is believed to be due, in part, to the property that the relative dielectric constant of the semiconductor is greater than that of the insulator. The normal derivative of the solution in the semiconductor that is specified at the interface is determined from that calculated in the insulator. This involves the scale factor, ε_2_/ε_1_ = (3.9/11.9) ≈ (1/3), that is less than one and serves to dampen the numerical error in the calculated solution. Physical arguments also show that this procedure brackets the normal derivative of the solution, and thus, the solution as well. A consistent solution is usually found within 10 iterations.

**Fig. 7:**
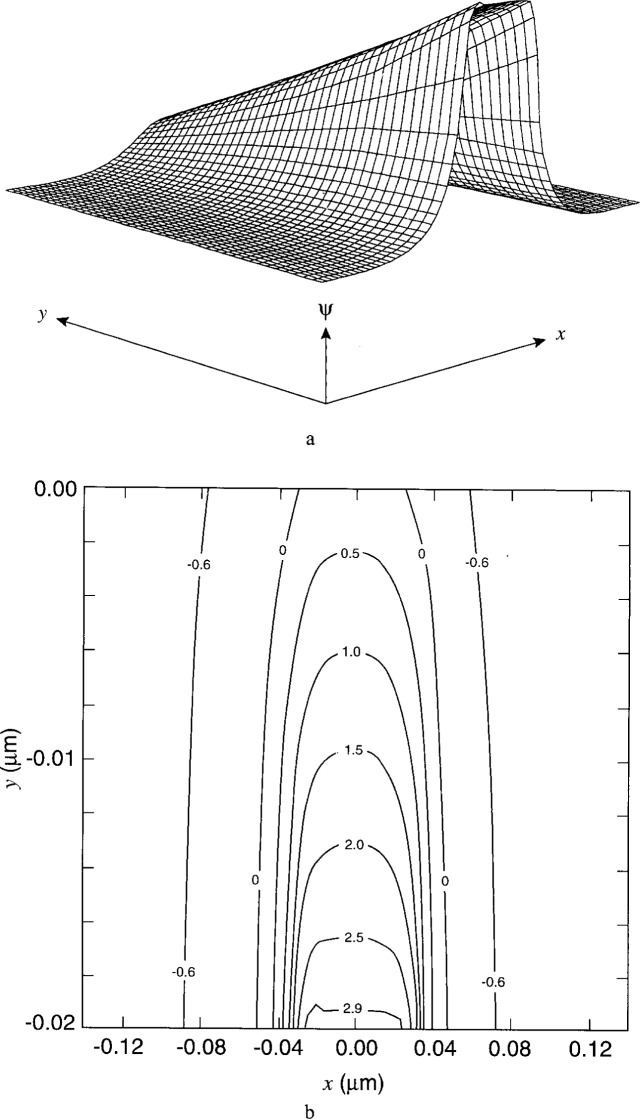
The potential distribution in the insulator region near the probe-tip region in the (*z* = 0) plane, where (−0.14 ≤ *x* ≤ 0.14), (−0.02 ≤ *y* ≤ 0), and (−0.76 ≤ *ψ* ≤ 3.0). (a) surface profile and (b) surface contours. The probe-tip bias is 3 V.

**Fig. 8:**
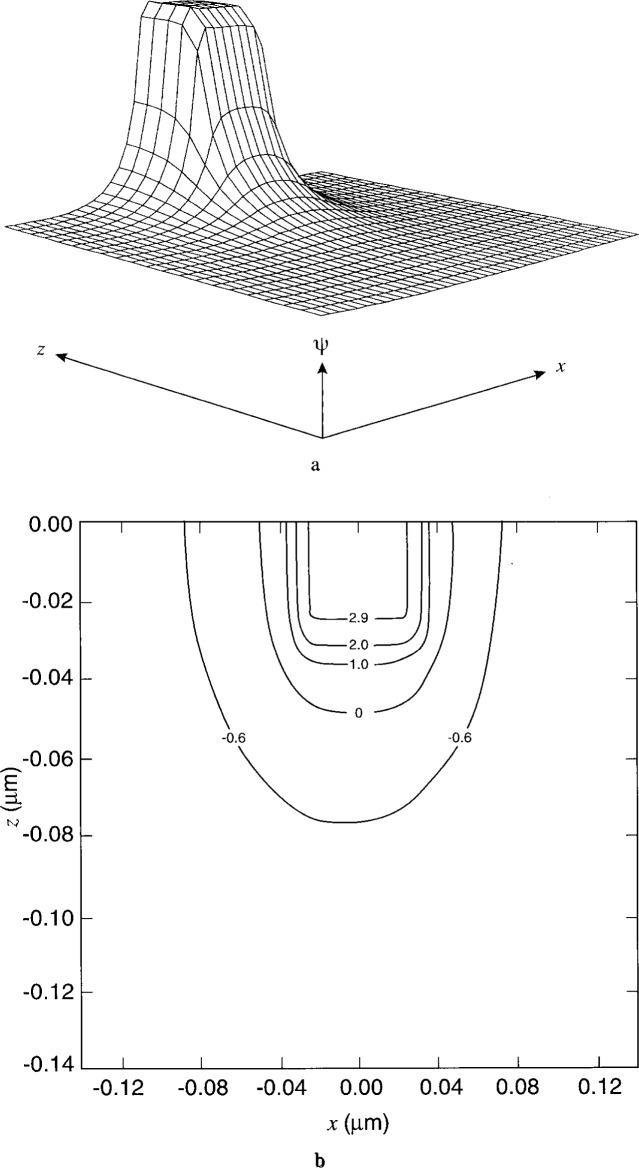
The potential distribution in the insulator region near the probe-tip region in the (*y* = −0.02) plane, where (−0.14 ≤ *x* ≤ 0.14), (−0.14 ≤ *z* ≤ 0), and (−0.76 ≤ *ψ* ≤ 3.0). (a) surface profile and (b) surface contours. The probe-tip bias is 3 V.

**Fig. 9:**
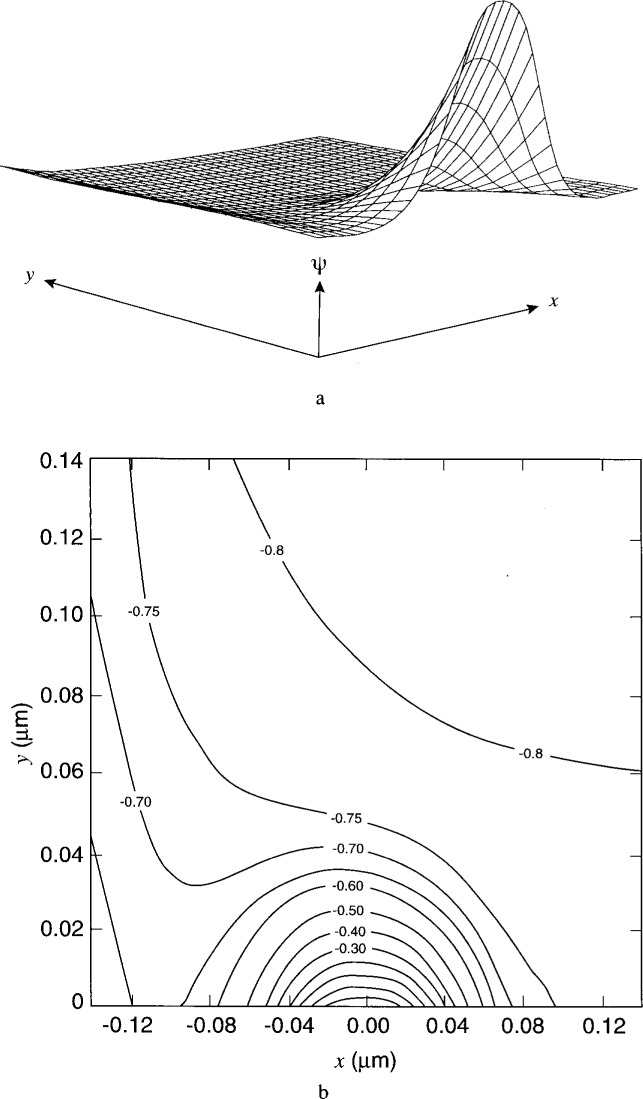
The potential distribution in the semiconductor region near the probe-tip region in the (*z* = 0) plane, where (−0.14 ≤ *x* ≤ 0.14), (0 ≤ *y* ≤ 0.14), and (−0.84 ≤ *ψ* ≤ 0.18). (a) surface profile and (b) surface contours.

**Fig. 10:**
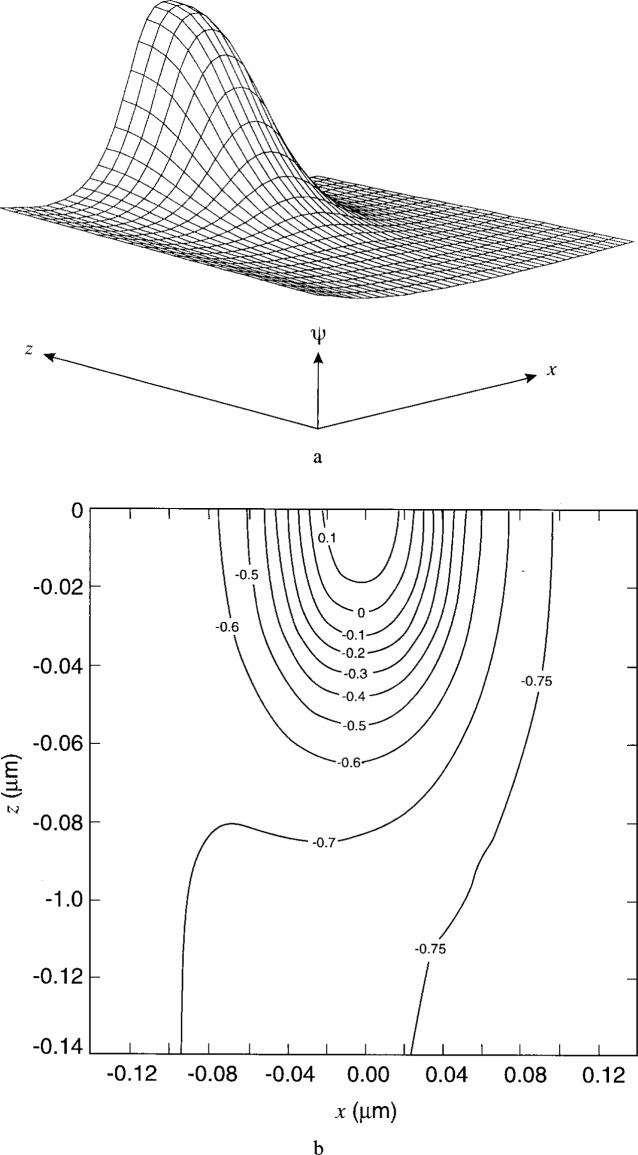
The potential distribution in the semiconductor region near the probe-tip region in the (*y* = 0) plane, where (−0.14 ≤ *x* ≤ 0.14), (−0.14 ≤ *z* ≤ 0.14), and (−0.76 ≤ *ψ* ≤ 0.18). (a) surface profile and (b) surface contours.

Figures 7 through 10 present a representative study of the results of calculation of the electric potential in the neighborhood near the biased probe-tip region. The probe-tip or patch region bias is 3 V. Figures 7a and 7b present the potential distribution in the insulator region in the (*z* = 0) plane. Figures 8a and 8b present the potential distribution in the insulator region in the (*y* = −0.02) plane. Figures 9a and 9b present the potential distribution in the semiconductor region in the (*z* = 0) plane. Figures 10a and 10b present the potential distribution in the semiconductor region in the (*y* = 0) plane.

Incidently, it may be worthwhile to comment on a more practical matter regarding the calculation, the matter of grid discretization. After it was found that the probe-tip induced a relatively localized perturbation on the unbiased steady-state equilibrium solution, one could then improve the representation of the solution near the probe-tip region if the model semiconductor problem were broken into two parts. Part 1 involved solving the unbiased steady-state equilibrium solution in the planar semiconductor region, where (−1 ≤ *x* ≤ 1) and (0 ≤ *y* ≤ 1). Part 2 involved solving the biased steady-state solution in a small volume region near and about the probe-tip. The size of this small probe-tip region was determined subjectively by requiring that it be a little larger than the size of the region that is being perturbed by the probe-tip, so that Dirichlet boundary conditions could be used to match the solution to the unbiased steady-state or equilibrium solution. The small probe-tip region was chosen to be given by (−0.14 ≤ *x* ≤ 0.14), (−0.02 ≤ *y* ≤ 0.14), and (−0.14 ≤ *z* ≤ 0). Dirichlet boundary conditions were used along the boundaries where (*x* = ±0.14), (*y* = 0.14), and (*z* = −0.14). After the solution was found, it was evaluated on a uniform grid over the small probe-tip region. These point function values were then used by the plotting package to form the plots that are presented in the figures. Transferring the solution between the two grids introduced small changes into the the final presentation of the solution. A more careful handling of the solution would eliminate these changes.

The asymmetry of the contours in Figs. 9b and 10b is due to the asymmetry of the ion-implanted dopant distribution about (*x* = 0). Compared to the smooth distribution near the origin that is presented in Fig. 5, Fig. 9b shows that the potential is locally perturbed from the equilibrium solution. The mobile electron distribution is attracted or displaced toward the probe-tip region when the probe-tip potential is above that of the equilibrium solution, which is certainly satisfied by being 3 V above the Fermi level. The characteristic size of the perturbation region is of the order of 0.1 μm. Since the differential capacitance is a measure of the displaced charge, the measurements of the differential capacitance would then be sensitive to the dopant concentration within this distance, i.e., 0.1 μm, from the surface. Here, a small perturbation region is important to the success of the differential capacitance technique in providing a sensitive nondestructive measurement of the surface dopant concentration.

## 5. Summary

A research code has been written to solve an elliptic system of coupled nonlinear partial differential equations of conservation form on a rectangularly shaped three-dimensional domain. The code uses the method of collocation of Gauss points with tricubic Hermite piecewise continuous polynomials as basis functions. The system of equations is solved by using iterative methods. The system of linear equations is solved by using software that implements the dual threshold incomplete LU factorization preconditioner, the look-ahead Lanczos algorithm, and the quasi-minimal residual method. When the matrix of the collocation equations is duly modified by using a scaled block-limited partial pivoting procedure of Gauss elimination, it is found that the rate of convergence of the iterative method is significantly improved and that a solution becomes possible. The code is used to solve a model semiconductor problem that is characteristic of a fabricated semiconductor wafer, a thermal oxide atop an ion-implanted semiconductor substrate. The model problem involves solving Poisson’s equation in two adjacent domain regions and the interface boundary conditions. For the given model semiconductor problem, it is found that the numerical solution has the correct limiting behavior to demonstrate the convergence of the software algorithms, and that the perturbed region in the semiconductor is of the order of 0.1 μm for the given dopant density and probe-tip size. A small perturbed region is important to the success of the differential capacitance technique in providing a sensitive nondestructive measurement of the surface dopant concentration.

## 6. Appendix A. Linearization of Equations

As discussed in Refs. [9,10,12], the standard procedure for solving a nonlinear coupled system of equations, like Eqs. (1) and (2), is to use Newton’s method, i.e., linearize the system in terms of the Newton step, solve the linear system for the Newton step, update the solution by the Newton step, and monitor the norm of the Newton step and determine whether to continue iterating. The same is followed here, as well.

Let the initial value solution, that is to be improved, be denoted by ***u*** with components *u_i_*. Let the Newton step, that is used to improve the solution and is to be determined, be denoted by ***v*** with components ***v****_i_*. To calculate ***v***, replace *u_i_* by *u_i_* + ***v****_i_* in Eqs. (1) and (2), and then linearize in ***v****_i_*.

Linearizing Eq. (1) yields

$$\begin{array}{c} \nabla \cdot \left[a_{i}\nabla v_{i}+(\nabla u_{i})\sum_{j=1}^{N}\frac{\partial a_{i}}{\partial u_{j}}v_{j}\right]\\ -\sum_{j=1}^{N}[\frac{\partial f_{i}}{\partial u_{j}}v_{j}+\frac{\partial f_{i}}{\partial (\partial u_{j}/\partial x)}\frac{\partial v_{j}}{\partial x}+\frac{\partial f_{i}}{\partial (\partial u_{j}/\partial y)}\frac{\partial v_{j}}{\partial y}+\\ \frac{\partial f_{i}}{\partial (\partial u_{j}/\partial z)}\frac{\partial v_{j}}{\partial z}]=f_{i}-\nabla \cdot [a_{i}\nabla u_{i}],\;i=1,2\ldots ,N, \end{array}$$ (A.1)

where the unknown variables ***v****_j_* have been collected on the left-hand side of the equation, and the residual is placed on the right-hand side. This equation is used for the interior collocation points.

Linearizing Eq. (2) yields

$$\begin{array}{c} \sum_{j=1}^{N}[\frac{\partial g_{i}}{\partial u_{j}}v_{j}+\frac{\partial g_{i}}{\partial (\partial u_{j}/\partial x)}\frac{\partial v_{j}}{\partial x}+\frac{\partial g_{i}}{\partial (\partial u_{j}/\partial y)}\frac{\partial v_{j}}{\partial y}+\\ \frac{\partial g_{i}}{\partial (\partial u_{j}/\partial z)}\frac{\partial v_{j}}{\partial z}]=-g_{i}. \end{array}$$ (A.2)

This equation is used for the boundary collocation points.

The PDEs are specified by using three user-supplied external subroutines, one for each function term, i.e., *a*, *f*, and *g*. These subroutines specify the PDEs by providing the coefficients in the terms that are linear in the unknown variables ***v****_j_* that are given in Eqs. (A.1) and (A.2).

The input arguments of the subroutines are:

(x,u,∂u/∂x,∂u/∂y,∂u/∂z). (A.3)

The output arguments of the subroutines depend on the function term.

For *a*, they are:

$$\begin{array}{r} (a_{i},\frac{da_{i}}{dx},\frac{da_{i}}{dy},\frac{da_{i}}{dz},\frac{da_{i}}{du_{j}},\\ \frac{d(\partial a_{i}/\partial u_{j})}{dx},\frac{d(\partial a_{i}/\partial u_{j})}{dy},\frac{d(\partial a_{i}/\partial u_{j})}{dz}), \end{array}$$ (A.4)

where

$$\frac{da_{i}}{dx}=\frac{\partial a_{i}}{\partial x}+\sum_{j=1}^{N}\frac{\partial a_{i}}{\partial u_{j}}\frac{\partial u_{j}}{\partial x},$$ (A.5)

$$\frac{d(\partial a_{i}/\partial u_{j})}{dx}=\frac{\partial (\partial a_{i}/\partial u_{j})}{\partial x}+\sum_{k=1}^{N}\frac{\partial (\partial a_{i}/\partial u_{j})}{\partial u_{k}}\frac{\partial u_{k}}{\partial x},$$ (A.6)

and derivatives of *y* and *z* follow analogously.

For *f*, they are:

$$\left(f_{i},\frac{\partial f_{i}}{\partial u_{j}},\frac{\partial f_{i}}{\partial (\partial u_{j}/\partial x)},\frac{\partial f_{i}}{\partial (\partial u_{j}/\partial y)},\frac{\partial f_{i}}{\partial (\partial u_{j}/\partial z)}\right).$$ (A.7)

For *g*, they are:

$$\left(g_{i},\frac{\partial g_{i}}{\partial u_{j}},\frac{\partial g_{i}}{\partial (\partial u_{j}/\partial x)},\frac{\partial g_{i}}{\partial (\partial u_{j}/\partial y)},\frac{\partial g_{i}}{\partial (\partial u_{j}/\partial z)}\right).$$ (A.8)

## 7. Appendix B. Basis Functions and Representation

As discussed in Ref. [11], the idea of collocation and osculating polynomials is to approximate a given function and its derivatives up to some order at specified arguments. The interest here is in approximating the solution *u_m_* over the finite-element mesh *Ω* of domain *D*, where *Ω* is formed by the tensor product of three one-dimensional finite-element meshes, i.e.,

$$\Omega =\underset{i,j,k}{\cup }\Omega_{i,j,k},$$ (B.1)

where *i*, *j*, and *k* index the mesh intervals of the one-dimensional finite-element meshes, that are used to form the tensor product volume element *Ω_i,j,k_*.

Within an interior element of a finite-element mesh [12], the approximate solution *u_m_*(*x,y,z*) is an Hermite tricubic piecewise continuous osculating polynomial that is defined on each element in terms of one-dimensional local basis functions, that have first-order osculation. The one-dimensional polynomial basis functions derived in terms of Lagrange multiplier functions are of the form [11]:

$$U_{i}(x)=\left[1-2L_{i}^{\prime }(x_{i})(x-x_{i})\right]{\left[\left(L_{i}(x\right)\right]}^{2},$$ (B.2)

$$V_{i}(x)=(x-x_{i}){\left[L_{i}(x)\right]}^{2},$$ (B.3)

where the prime indicates a derivative, *i* = {0,1} indexes the distinct grid points *x_i_*, (*x*_0_ ≤ *x* ≤ *x*_1_), and *L_i_*(*x*) refers to the two-point Lagrange multiplier functions that are given by:

$$L_{0}(x)=(x_{1}-x)/(x_{1}-x_{0}),$$ (B.4)

$$L_{1}(x)=(x-x_{0})/(x_{1}-x_{0}).$$ (B.5)

These functions have the property that:

$$L_{i}(x_{j})=\delta_{ij},$$ (B.6)

$$U_{i}(x_{j})=\delta_{ij},\;U_{i}^{\prime }(x_{j})=0,$$ (B.7)

$$V_{i}(x_{j})=0,\;V_{i}^{\prime }(x_{j})=\delta_{ij},$$ (B.8)

where *δ* refers to the Kronecker delta,

$$\delta_{ij}=\{\begin{array}{ll} 1, & \text{if}\;i=j;\\ 0, & \text{otherwise} \end{array}$$ (B.9)

The approximating polynomial in one dimension is of the form:

$$p(x)=\sum_{i=0}^{1}\left\{U_{i}(x)f(x_{i})+V_{i}(x)f_{x}(x_{i})\right\};$$ (B.10)

the approximating polynomial in two dimensions is of the form:

$$\begin{array}{c} p(x,y)=\sum_{i=0}^{1}\sum_{j=0}^{1}\left\{U_{i}(x)U_{j}(y)f(x_{i},y_{j}\right)\\ +V_{i}(x)U_{j}(y)f_{x}(x_{i},y_{j})\\ +U_{i}(x)V_{j}(y)f_{y}(x_{i},y_{j})\\ +V_{i}(x)V_{j}(y)f_{xy}(x_{i},y_{j}); \end{array}$$ (B.11)

and the approximating polynomial in three dimensions is of the form:

$$\begin{array}{c} p(x,y,z)=\sum_{i=0}^{1}\sum_{j=0}^{1}\sum_{k=0}^{1}\left\{U_{i}(x)U_{j}(y)U_{k}(z)f(x_{i},y_{j},z_{k}\right)\\ +V_{i}(x)U_{j}(y)U_{k}(z)f_{x}(x_{i},y_{j},z_{k})\\ +U_{i}(x)V_{j}(y)U_{k}(z)f_{y}(x_{i},y_{j},z_{k})\\ +U_{i}(x)U_{j}(y)V_{k}(z)f_{z}(x_{i},y_{j},z_{k})\\ +U_{i}(x)V_{j}(y)V_{k}(y)f_{yz}(x_{i},y_{j},z_{k})\\ +V_{i}(x)U_{j}(y)V_{k}(z)f_{xz}(x_{i},y_{j},z_{k})\\ +V_{i}(x)V_{j}(y)U_{k}(z)f_{xy}(x_{i},y_{j},z_{k})\\ +V_{i}(x)V_{j}(y)V_{k}(z)f_{xyz}(x_{i},y_{j},z_{k})\}; \end{array}$$ (B.12)

where (*x*_0_ ≤ *x* ≤ *x*_1_), (*y*_0_ ≤ *y* ≤ *y*_1_), (*z*_0_ ≤ *z* ≤ *z*_1_), and the subscripts on the function *f* refer to partial derivatives. Given these forms, it follows that approximate solution *u_m_*(*x*,*y*,*z*) is the same form as Eq. (B.12), except that now the point functions *f*, are replaced by coefficients that are to be determined at the mesh points of the partition *Ω*. Thus, one seeks to determine the values of a function and its accompanying set of partial derivatives at each mesh point. This yields a set of eight values per mesh point for each solution component or partial differential equation that is to be solved.

The coefficients of the solution are collected together in an array that is called the collocation vector. The array is organized according to the form implied by (*n_b_*,*n_u_*,*n_x_*,*n_y_*,*n*_z_), where the dimensioning and indexing convention of Fortran is used, *n_b_* refers to the number of basis functions, i.e., 8; *n_u_* refers to the number of components to the solution, i.e., *N* PDEs; *n_x_* refers to the number of mesh points associated with the *x*-axis; *n_y_* refers to the number of mesh points associated with the *y*-axis; and *n_z_* refers to the number of mesh points associated with the *z*-axis. The coefficients associated with one mesh point and one solution component is collectively called the local collocation vector, and the coefficients are ordered in a manner that is suggested by Eq. (B.12), i.e., (*u*,*u_x_*,*u_y_*,*u_z_*,*u_yz_*,*u_zx_*,*u_xy_*,*u_xyz_*). The local collocation vector of the Newton step is of the form: (***v***, ***v****_x_*, ***v****_y_*, ***v****_z_*, ***v****_yz_*, ***v****_zx_*, ***v****_xy_*, ***v****_xyz_*); see Appendix A.
